# The Ablation of VEGFR-1 Signaling Promotes Pressure Overload-Induced Cardiac Dysfunction and Sudden Death

**DOI:** 10.3390/biom11030452

**Published:** 2021-03-17

**Authors:** Annakaisa Tirronen, Nicholas L. Downes, Jenni Huusko, Johanna P. Laakkonen, Tomi Tuomainen, Pasi Tavi, Marja Hedman, Seppo Ylä-Herttuala

**Affiliations:** 1 A.I. Virtanen Institute for Molecular Sciences, University of Eastern Finland, 70211 Kuopio, Finland; annakaisa.tirronen@uef.fi (A.T.); nicholas.downes@uef.fi (N.L.D.); jenni.huusko@novartis.com (J.H.); johanna.p.laakkonen@uef.fi (J.P.L.); tomi.tuomainen@uef.fi (T.T.); pasi.tavi@uef.fi (P.T.); 2 Institute of Clinical Medicine, University of Eastern Finland, 70029 Kuopio, Finland; marja.hedman@kuh.fi; 3 Heart Center and Cardiothoracic Surgery, Kuopio University Hospital, 70029 Kuopio, Finland; 4 Heart Center and Gene Therapy Unit, Kuopio University Hospital, 70029 Kuopio, Finland

**Keywords:** VEGFR-1, Flt1, heart failure, HFpEF, hypertrophy, pressure overload

## Abstract

Molecular mechanisms involved in cardiac remodelling are not fully understood. To study the role of vascular endothelial growth factor receptor 1 (VEGFR-1) signaling in left ventricular hypertrophy (LVH) and heart failure, we used a mouse model lacking the intracellular VEGFR-1 tyrosine kinase domain (VEGFR-1 TK^−/−^) and induced pressure overload with angiotensin II infusion. Using echocardiography (ECG) and immunohistochemistry, we evaluated pathological changes in the heart during pressure overload and measured the corresponding alterations in expression level and phosphorylation of interesting targets by deep RNA sequencing and Western blot, respectively. By day 6 of pressure overload, control mice developed significant LVH whereas VEGFR-1 TK^−/−^ mice displayed a complete absence of LVH, which correlated with significantly increased mortality. At a later time point, the cardiac dysfunction led to increased ANP and BNP levels, atrial dilatation and prolongation of the QRSp duration as well as increased cardiomyocyte area. Immunohistochemical analyses showed no alterations in fibrosis or angiogenesis in VEGFR-1 TK^−/−^ mice. Mechanistically, the ablation of VEGFR-1 signaling led to significantly upregulated mTOR and downregulated PKCα phosphorylation in the myocardium. Our results show that VEGFR-1 signaling regulates the early cardiac remodelling during the compensatory phase of pressure overload and increases the risk of sudden death.

## 1. Introduction

Heart failure has been estimated to affect approximately 1–2% of the adult population in developed countries [1]. The continuous prolongation of the mean life expectancy and the increase in additional risk factors such as obesity, coronary artery disease and hypertension are the main attributes to the high incidence. Compared to the other major forms of cardiovascular disease, heart failure is the only disease with increasing prevalence and mortality [2]. Patients with heart failure commonly have a history of hypertension and consequent left ventricular hypertrophy (LVH) [3]. When pressure overload is sustained, cardiac remodelling occurs, as an adaptive response, which includes development of LVH combined with diastolic and/or systolic dysfunction. Eventually this leads to decompensated hypertrophy characterised by contractile dysfunction, reduced coronary flow reserve, interstitial fibrosis, as well as changes in metabolism and electrophysiology, triggering clinically evident heart failure.

Heart failure can be classified according to the LV systolic function measured as LV ejection fraction. This classification includes heart failure with preserved ejection fraction (HFpEF with EF over 50%) and heart failure with reduced ejection fraction (HFrEF with EF less than 40%). Both HFpEF and HFrEF patients have equally poor prognosis, the 5 year absolute all-cause mortality is around 55% [4]. The molecular mechanisms leading to cardiac remodelling and heart failure are not fully understood but defective angiogenesis is one factor promoting the transition from compensatory LVH to decompensated heart failure [4,5]. Heart requires adequate oxygen and nutrition supply to maintain myocyte growth during hypertrophy and therefore insufficient blood flow and angiogenesis could result in contractile dysfunction and accelerated progression from compensatory cardiac hypertrophy to heart failure.

Vascular endothelial growth factor receptor 1 (VEGFR-1) belongs to the VEGF-receptor family, and mediate signalling of VEGFs, which are important mediators of vasculogenesis, angiogenesis and vascular maintenance. VEGFR-1 specific ligands, VEGF-B and placental growth factor (PlGF), have been shown to exhibit angiogenic properties in the myocardium [6,7,8]. However, several studies suggest that the main function of VEGFR-1 is to sequester VEGF-A and thus modulate VEGFR-2 signaling and angiogenesis [9,10,11]. VEGFR-1 knockout mice die in utero due to overgrowth and disorganisation of endothelial cells whereas VEGFR-1 tyrosine kinase (TK)-deficient mice are healthy and fertile [12,13]. Additionally, VEGFR-1 has an approximately 10-fold higher binding affinity to VEGF-A than VEGFR-2, but its kinase activity is very weak [13]. Vascular growth factors have been shown to induce myocardial hypertrophy in mice and rats, but the signaling mechanisms that mediate this growth are poorly understood. VEGF-B expression is downregulated in heart failure and VEGF-B gene therapy has been shown to have a protective role in progressive LVH [14]. However, it remains unknown whether VEGFR-1 signaling is essential in pathological LVH.

In this study, we investigated whether VEGFR-1 signaling has a role in the progression of LVH and heart failure. We also studied what molecular changes the deficiency of VEGFR-1 signaling induces in the myocardium and whether it has a role in cardiac angiogenesis. For this we used domain-specific knockout mouse lacking the intracellular VEGFR-1 tyrosine kinase domain (TK^−/−^) and induced pathological hypertrophy with angiotensin II infusion and followed the disease progression. Here we show that the ablation of VEGFR-1 signaling halts the development of compensatory hypertrophy during pressure overload and leads to significantly increased mortality. Furthermore, VEGFR-1 TK^−/−^ mice exhibited increased ANP and BNP levels and showed significant alterations in echocardiography (ECG) but no changes in myocardial angiogenesis or fibrosis. Protein expression analyses revealed significantly upregulated mTOR and downregulated PKCα phosphorylation in the myocardium, suggesting that VEGFR-1 not only functions as a decoy receptor, but its signaling has a significant role in the development of pathological LVH and prevention of heart failure.

## 2. Materials and Methods

### 2.1. Angiotensin II Infusion

Pathological LVH was generated by angiotensin II (angII, Phoenix Pharmaceuticals Inc., CA, USA) infusion via subcutaneous Alzet osmotic minipumps (Durect Corporation, Cupertino, CA, USA) for two weeks (0.1 mg/kg/h). Five-to-seven-month-old male VEGFR-1 TK^−/−^ mice [12] and their littermate controls were anesthetised with isoflurane (induction: 4.5% isoflurane, 450 mL air, maintenance: 2.0% isoflurane, 200 mL air, Baxter 28 International Inc., Deerfield, IL, USA) for the operation and an analgesic (carprofen 5 mg/kg, s.c.; Rimadyl, Pfiser Inc., New York, NY, USA) was administered. The mice were kept in standard housing conditions in the National Laboratory Animal Centre of the University of Eastern Finland. Diet and water were provided ad libitum. Mice were euthanized with CO_2_ and perfused with phosphate-buffered saline (PBS). All animal procedures were approved by the Animal Experiment Board in Finland and were carried out in accordance with the guidelines of the Experimental Animal Committee of the University of Eastern Finland. The animal licence number is ESAVI-2014-5089.

### 2.2. Echocardiography and Electrocardiography Recording

High-resolution transthoracic echocardiographic measurements were performed using VEVO2100 Ultrasound System for small animals (Fujifilm VisualSonics Inc., Toronto, ON, Canada) using a high-frequency ultrasound probe (MS400) operating at 18–38 MHz. Mice were anesthetised with isoflurane induction: 4.5% isoflurane, 450 mL air and maintenance; 2.0% isoflurane, 200 mL air (Baxter 28 International Inc., Deerfield, IL, USA). LV dimensions and wall thickness were measured from the parasternal short axis M-Mode images using papillary muscles as an indication of mid-ventricular level. LV systolic function was measured by using Teicholz method and diastolic function by measuring the LV relaxation time starting from the peak systole and ending when the maximum relaxation of LV posterior wall was achieved. The analysis was conducted from three consecutive cardiac cycles using VEVO Lab software (Fujifilm VisualSonics Inc., ON, Canada). In addition, ECG signal was acquired via limb electrodes during echocardiography and data were analysed with rodent ECG imaging software (Kubios HRV 2.0, Kuopio, Finland) as previously described [15].

### 2.3. Immunohistochemistry

For immunohistochemical analyses, 4 µm thick paraffin-embedded cardiac sections fixed with 4% paraformaldehyde were used. Haematoxylin-Eosin staining was performed to characterise tissue morphology and anti-laminin antibody (ab11575, 1:500, Abcam, Cambridge, UK) with goat anti-rabbit IgG Alexa 594 (Thermo Fisher Scientific, Waltham, MA, USA) were used to visualise the cardiomyocyte area. To analyse hypertrophy-induced fibrosis, cardiac sections were stained with Masson´s trichrome (Sigma-Aldrich, St Louis, MO, USA) and Picro Sirius Red (Abcam, Cambridge, UK). Blood capillaries were stained with biotinylated lectin (Biotinylated Griffonia (Bandeiraea) Simplicifolia Lectin I, 1:100, Vector Laboratories, Burlingame, CA, USA). αSMA-positive vessels were detected with αSMA antibody (anti-alpha smooth muscle actin-Cy3, 1:200, Sigma-Aldrich, St Louis, MO, USA) and VEGFR-1 with mouse VEGFR-1 antibody (AF471, 1:200, R&D, Minneapolis, MN, USA). Apoptotic cells were detected with Click-iT™ Plus Tunel assay (Thermo Fisher Scientific, Waltham, MA, USA). Tissue sections were imaged with a light microscope (Nikon Eclipse Ni, Nikon, Tokyo, Japan) and image analyses were performed with ImageJ software.

For visualisation of the myocardial capillary network, PFA-fixed myocardial tissue was sliced about 1 mm thick and immersed in Immunomix (0.3% Triton-X, 0.2% BSA and 5% normal serum in PBS). Rat anti-mouse CD31 (553370, BD Pharmingen, Franklin Lakes, NJ, USA)) was added and tissue samples were incubated overnight. Then samples were washed with PBS and incubated with goat anti-rat IgG Alexa 594 (Thermo Fisher Scientific, Waltham, MA, USA) overnight. Imaging was performed with Zeiss LSM800 (Carl Zeiss, Oberkochen, Germany) Airyscan confocal laser scanning microscope and 555 nm diode laser was used together with the appropriate emission filter. 10×/0.3 or 20×/0.5 PlanApo objectives were used (1024 × 1024 frame size). Maximum intensity projections were generated with ImageJ software [16].

### 2.4. Western Blotting

Proteins from snap frozen cardiac tissues were extracted using T-PER Tissue Protein Extraction Reagent (Thermo Fisher Scientific, Waltham, MA, USA) and total protein concentration was determined by BCA kit (Thermo Fisher Scientific, Waltham, MA, USA). Then, 20–40 µg of protein were separated on Mini-PROTEAN TGX Stain-free gels (Bio-Rad, Hercules, CA, USA) and transferred to nitrocellulose membrane (Bio-Rad, Hercules, CA, USA). The membranes were incubated in iBind automated western system (Invitrogen, Waltham, MA, USA) with primary antibodies against VEGFR-2 (55B11, Cell Signaling, Danvers, MA, USA), VEGF-A (sc7269 Santa Cruz Biotechnology, Dallas, TX, USA), Akt (9272, Cell Signaling, Danvers, MA, USA), p-Akt (4058, Cell Signaling, Danvers, MA, USA), mTOR (2983, Cell Signaling, Danvers, MA, USA), p-mTOR (5536, Cell Signaling, Danvers, MA, USA), PKG-1 (3248, Cell Signaling, Danvers, MA, USA), PKCα (ab32376, Abcam, Cambridge, UK), p- PKCα (ab76016, Abcam, Cambridge, UK), ERK1/2 (137F5, Cell Signaling, Danvers, MA, USA), p-ERK1/2 (20G11, Cell Signaling, Danvers, MA, USA) and GAPDH (2118, Cell Signaling, Danvers, MA, USA) (1:1000). Stain-free gels were UV-activated for 2.5 min before blotting and total protein amount was measured with ChemiDox XRS (Bio-Rad, Hercules, CA, USA) before enhanced chemiluminescent (ECL) detection. Specific bands were normalised to total protein or GAPDH expression using ImageLab software (Bio-Rad, Hercules, CA, USA).

### 2.5. RNA Isolation and RT-qPCR

Adult mouse cardiac cells were isolated with a Langendorff perfusion system as previously described [17]. Immediately after excision, cardiac tissue was placed in Langendorff apparatus and tissue break-down was performed with trypsin (Sigma-Aldrich, St Louis, MO, USA) and liberase (Roche Applied Science, Mannheim, Germany) enzymes. Ventricles were cut into small pieces and single-cell suspension was achieved by carefully triturating with glass Pasteur pipette. Cell suspension was centrifuged for 1 min at 700 g, and pelleted cells were collected as a cardiomyocyte sample and supernatant as a sample containing other cell types. Total RNA from adult cardiomyocytes and heart tissue was isolated using TRI Reagent Solution (Invitrogen, Carlsbad, CA, USA) according to the manufacturer’s recommendations. Then, 1.5 µg of RNA, purified from the aqueous phase with RNeasy Mini Spin columns (Qiagen, Hilden, Germany), was used for cDNA synthesis. RNA was reverse transcribed by using random hexamer primers and RevertAid Reverse Transcriptase (Thermo Scientific, Waltham, MA, USA) and gene expression was analysed using PrimeTime qPCR primer-probe sets (IDT, Newark, NJ, USA) or TaqMan gene expression assays (Thermo Fisher Scientific, Waltham, MA, USA). Quantitative measurements of mRNAs were performed with StepOnePlus Real-Time PCR System (Thermo Fisher Scientific, Waltham, MA, USA). The catalog numbers for gene expression assays: Col3a1 (Mm.PT.58.13848686), NPPA (Mm01255747_g1), NPPB (Mm01255770_g1), PPIA (Mm02342430_g1), RPLP0 (Mm.PT.5843894205), VEGF-B (Mm01231781_g1), VEGFR-1 (Mm.PT.5843852013).

### 2.6. RNA Sequencing

Total RNA was isolated with TRI Reagent Solution (Invitrogen) according to the manufacturer’s recommendations. The yield and integrity (RIN > 7.0) for each of the isolated RNA samples were assessed using Agilent 2100 Bioanalyzer (Agilent Technologies, Santa Clara, CA, USA), respectively. Next, 1 µg of RNA per sample was sent to Macrogen (Seoul, South Korea) for library construction and sequencing. Poly-A-selected RNA was used to prepare cDNA libraries with the TruSeq stranded mRNA library prep Kit (Illumina, San Diego, CA, USA). Libraries were QC checked and quantitated using the Agilent 2100 Bioanalyzer (Agilent Technologies, Santa Clara, CA, USA) and equimolar pooled and paired-end sequenced (2 × 100 cycles) on the NovaSeq 6000 (Illumina, San Diego, CA, USA) for 30 million reads per sample. Paired-end reads were trimmed and filtered using Trim Galore (v.0.4.4) with Phred quality score cutoff 30. Processed reads were aligned to the genome assembly GRCm38 using STAR version 2.5.4b [18] with options --outFilterMismatchNoverLmax 0.04 and --outFilterMultimapNmax 10. Aligned reads mapping to features were assigned using featureCounts (Rsubread 1.32.4) with the sGencode M16 GTF. Transcripts with low counts of CPM < 1 not present in at least 2 libraries above this threshold were considered not expressed and removed from the analysis. Library sizes were subsequently normalised using trimmed mean of M-values (TMM) and the generalised linear model likelihood ratio test was used to estimate differential expression using edgeR (3.24.3). Transcripts with a log FC of ≥ 0.585 or ≤ −0.585 and *FDR* < 0.1 were considered differentially expressed. Plots were produced and visualised in R using ggplot2. Multidimensional scaling (MDS) was performed using cmdscale.

### 2.7. Data Availability

The RNA sequencing data generated in this publication have been deposited in GEO under the accession GSE162489.

### 2.8. Statistical Analysis

Statistical analyses were performed with GraphPad Prism v5.03 (GraphPad Software). Significance was tested using Student’s *t*-test or one-way ANOVA with Bonferroni´s Multiple comparison post-test. Survival curves were created using the Kaplan–Mayer method and survival curves were compared with a log-rank test. Results are expressed as mean ± SEM and differences were considered statistically significant at * *p* < 0.05, ** *p* < 0.01, *** *p* < 0.001.

## 3. Results

### 3.1. Deletion of the VEGFR-1 Signaling Halts the Early Development of Myocardial Compensatory Hypertrophy and Leads to Cardiac Dysfunction

**Figure 1:**
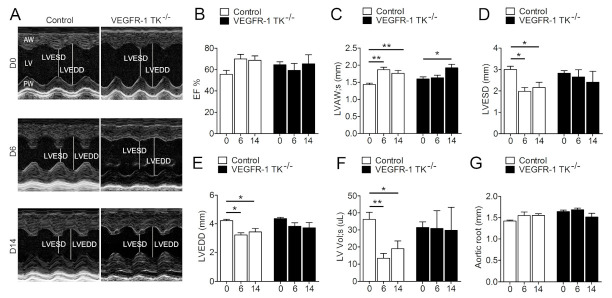
VEGFR-1 signaling regulates the early stage of compensatory LVH. Progression of LVH as followed by echocardiography. (**A**) Representative short axis view images of LVs at day 0, 6 and 14. (**B**) LV ejection fraction (EF; n = 6–13/group). (**C**) LV anterior wall (LVAW) thickness (n = 6–13/group). (**D**). LV end-systolic diameter (LVESD; n = 6–13/group). (**E**) LV end-diastolic diameter (LVEDD; n = 6–13/group). (**F**) LV systolic volume (LV Vol;s; n = 6–13/group). (**G**) Aortic root diameter (n = 6–13/group). Values are mean ± SEM. Statistical analyses were performed using one-way ANOVA with Bonferroni’s post hoc test. *** *p* < 0.001; ** *p* < 0.01; * *p* < 0.05.

Several publications have shown that VEGFR-1 ligands VEGF-A and VEGF-B in the heart induce LVH and angiogenesis as well as regulate cardiac function [7,8,19]. To test whether VEGFR-1 signaling is involved in these processes, we studied cardiac function at baseline and during pressure overload using domain–specific knockout mouse lacking the intracellular VEGFR-1 tyrosine kinase domain (TK^−/−^). First, we performed baseline cardiac function analysis (day 0) with high-resolution transthoracic echocardiography for five-to-seven-month-old VEGFR-1 TK^−/−^ mice and their littermate control mice. Both groups displayed similar cardiac parameters indicating that VEGFR-1 TK activity is not required for normal cardiac function at resting conditions (Figure 1A–F).

To assess the effects of VEGFR-1 signaling in the development of LVH, we inserted subcutaneous angII pumps in VEGFR-1 TK^−/−^ mice and their littermate controls. Mice were followed by echocardiography for two weeks to evaluate the cardiac function and the progression of LVH induced by pressure overload. At day 6 of pressure overload, control mice exhibited rapid cardiac remodelling and the development of compensatory LVH was seen as a 20.6% increase in ejection fraction (EF) and significantly elevated thickness of the anterior wall of the left ventricle (LVAW) and decreased end-systolic as well as end-diastolic diameters (Figure 1B–E). In addition, left ventricular systolic volume (LV Vol;s) was significantly reduced (Figure 1F). In contrast, VEGFR-1 TK^−/−^ mice displayed no response to pressure overload and showed no changes in the cardiac parameters at day 6 (Figure 1B–F). However, VEGFR-1 TK^−/−^ mice had a LV filling dysfunction seen as a slower LV relaxation in M-mode ultrasound recording compared to control mice (Figure 1A) (62.5% vs. 16.7% of mice, respectively). At day 14, VEGFR-1 TK^−/−^ mice showed a significant thickening of the LVAW, whereas the LV volume and EF remained unaltered (Figure 1B,C,F). Aortic root diameter did not change during pressure overload in either of the two groups (Figure 1G).

**Figure 2:**
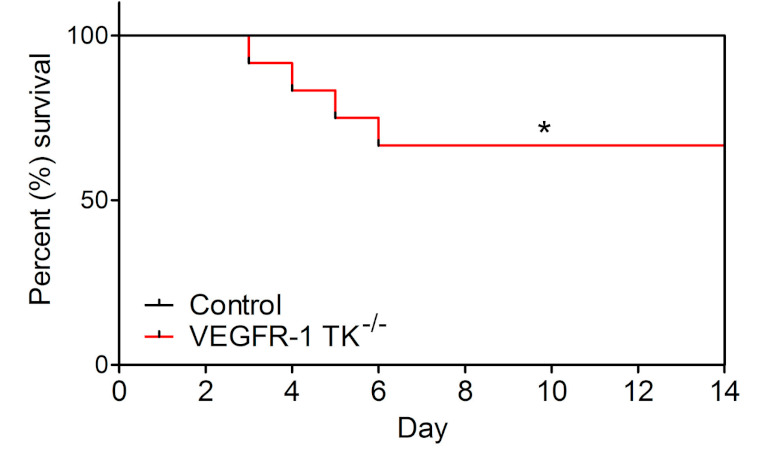
VEGFR-1 TK^−/−^ mice show significantly increased mortality. Mortality of VEGFR-1 TK^−/−^ and control mice during 14-day follow-up after angII infusion (n = 10–13/group). Values are mean ± SEM. Statistical analysis was performed using Kaplan–Mayer with log-rank test for survival curve. *** *p* < 0.001; ** *p* < 0.01; * *p* < 0.05.

Unexpectedly, pressure overload had an effect on VEGFR-1 TK^−/−^ mice mortality by reducing their survival by 33.3% compared to the controls (Figure 2). Mortality was the highest on days 3–6 of angII infusion in parallel to the absence of compensatory LVH development indicating the lack of pressure overload-induced cardiac remodelling in these mice. 

**Figure 3:**
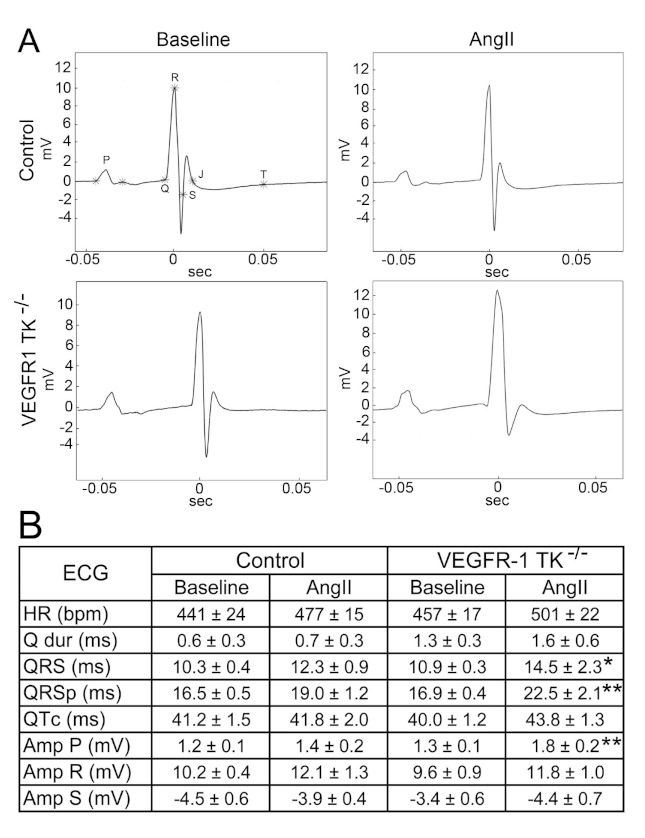
Deletion of VEGFR-1 signaling leads to QRSp prolongation after pressure overload. (**A**) Representative electrocardiographs of baseline and day 14 of angII infusion. (**B**) Electrocardiographic measurements during baseline and LVH (n = 7–13/group). Values are mean ± SEM. Statistical analyses were performed using Student´s *t*-test. *** *p* < 0.001; ** *p* < 0.01; * *p* < 0.05. Amp indicates amplitude; BPM, beats per minute; HR, heart rate; ms, millisecond; mV millivolt.

We acquired surface ECG signal during echocardiographic imaging and were able to show that, at day 14, LVH induces a significant prolongation in ventricular depolarisation of VEGFR-1 TK^−/−^ mice, which is seen as widening of the QRS complex by 25% (Figure 3A,B). Additionally, QRSp duration was also prolonged by 25% representing an increase in ventricular depolarisation and early repolarisation. There were no significant changes in R or S waves; however, both groups exhibited some changes in J wave, including widening and lowering of J wave. The amplitude of P wave was increased in VEGFR-1 TK^−/−^ mice, suggesting atrial dilatation. No arrhythmias were detected during the ECG recordings.

Altogether, VEGFR-1 signaling is required for the early cardiac remodelling induced by pressure overload, and the loss of signaling results in the lack of compensatory LVH at early time point and the higher pressure overload-induced sudden cardiac death rate. Furthermore, VEGFR-1 TK deletion induced significant alterations in ECG such as prolongation of QRSp, increase in the amplitude of P wave and causes cardiac dysfunction.

### 3.2. VEGFR-1 Tyrosine Kinase Deficiency Results in a Delayed but more Robust Development of Cardiomyocyte Hypertrophy

**Figure 4:**
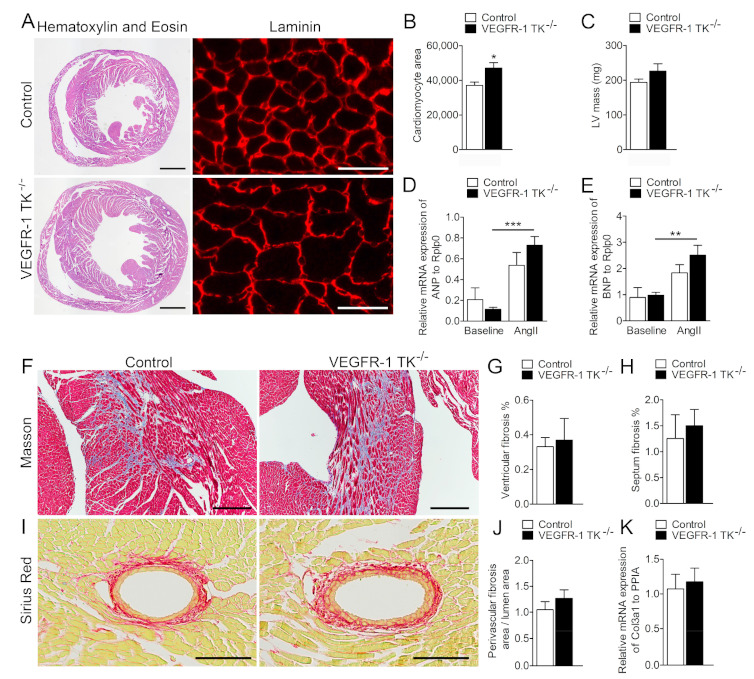
VEGFR-1 TK^−/−^ mice display cardiomyocyte hypertrophy at day 14. (**A**) Haematoxylin & Eosin (scale bar is 1000 µm) and laminin (scale bar is 25 µm) stainings of the myocardium at day 14 of angII infusion. (**B**) Quantification of cardiomyocyte area (n = 8–10/group). (**C**) LV mass measured with echocardiogram (n = 7–11/group). (**D**) Relative ANP mRNA expression measured with RT qPCR (n = 5–7/group). (**E**) Relative BNP expression measured with RT qPCR (n = 5–7/group). (**F**) Masson’s staining of the myocardium (scale bar is 250 µm). (**G**) Quantification of the level of fibrosis in the ventricles (n = 6–9/group). (**H**) Quantification of the level of septal fibrosis (n = 6–9/group). (**I**) Sirius Red staining of the perivascular fibrosis (scale bar is 100 µm). (**J**) Quantification of the level of perivascular fibrosis (n = 6–9/group). (**K**) Relative mRNA expression of Col3a1 measured with RT qPCR (n = 5–7/group). Values are mean ± SEM. Statistical analyses were performed using one-way ANOVA with Bonferroni’s post hoc test and Student´s *t*-test. *** *p* < 0.001; ** *p* < 0.01; * *p* < 0.05.

After 14 days of angII infusion, we collected tissue samples from the surviving mice and performed histological, transcriptomics and protein analyses. To compare the effects of VEGFR-1 TK deletion after angII infusion in histological level, we visualised cardiac tissue morphology and assessed putative structural changes with Haematoxylin-Eosin and laminin staining from day 14 time point. In cross sections, VEGFR-1 TK^−/−^ mice exhibited significantly larger cardiomyocyte area (Figure 4A), that was confirmed by cell area quantification (21% increase compared to the controls) and echocardiographic measurement of LV mass (15% increase compared to the controls) revealing more robust cardiac hypertrophy in VEGFR-1 TK^−/−^ mice compared to the control mice (Figure 4B,C).

ANP and BNP secretion from the heart is increased during hemodynamic overload and cardiac remodelling both in humans and in experimental animal models [5]. ANP and BNP levels were increased after angII infusion, in particular in VEGFR-1 TK KO mice, suggesting cardiac dysfunction (Figure 4D,E). Cardiac fibrosis is a key pathological process in LVH and cardiac dysfunction, and it can be seen as diffuse, disproportionate accumulation of collagen in the myocardial interstitium. Interstitial fibrosis was graded from Masson’s trichrome and perivascular fibrosis from Sirius Red-stained sections, but no significant differences in collagen deposition in VEGFR-1 TK^−/−^ and control mice were observed, and in addition *Col3a1* mRNA levels were similar in both groups (Figure 4F–K).

### 3.3. Deletion of VEGFR-1 Signaling Has no Effect on Angiogenesis in the Myocardium 

**Figure 5:**
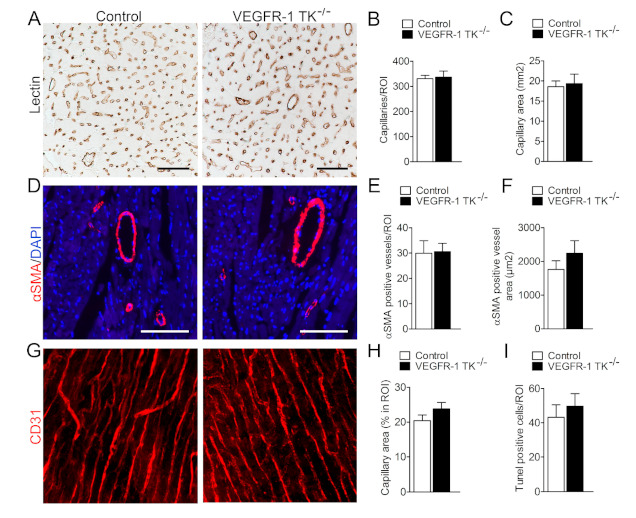
VEGFR-1 TK signaling does not affect angiogenesis. (**A**) Representative images of lectin staining of myocardial sections at day 14 of angII infusion (scale bar is 50 µm). (**B**) Quantification of capillary density in LV (n = 7–9/group). (**C**) Quantification of capillary area (n = 7–9/group). (**D**) Representative images of staining for arteries (αSMA; scale bar is 100 µm). (**E**) Quantification of arterial vessel density (n = 8–10/group). (**F**) Quantification of αSMA-positive vessel area (n = 8–10/group). (**G**) Representative images of staining for CD31 to visualise capillary tree at baseline. (**H**) Quantification of capillary area (n = 6–9/group). (**I**) Quantification of apoptotic cells at day 14 of angII infusion (n = 8–10/group). Values are mean ± SEM. Statistical analyses were performed using Students *t*-test. *** *p* < 0.001; ** *p* < 0.01; * *p* < 0.05.

Chronic pressure overload triggers physiological angiogenesis to maintain adequate oxygen and nutrition flow to growing heart muscle. Since VEGFR-1 is a known regulator of angiogenesis, we wanted to assess the potential angiogenic changes induced by VEGFR-1 TK signaling during angII infusion. For this we measured the level of angiogenesis and the cross-sectional area of capillaries in both VEGFR-1 TK^−/−^ and control mice at day 14 time point. Histological sections were lectin-stained to visualise ventricular capillary cross-sectional area and αSMA-stained to assess arterial density as well as area. VEGFR-1 TK^−/−^ mice showed no impairment in cardiac angiogenesis during pathological hypertrophy, both capillary and arterial cross-sectional area and density remained unaltered (Figure 5A–F). 3D capillary hierarchy of baseline mice was visualised with pericardial CD31 staining combined with confocal microscopy (Figure 5G). The capillary network was similar between both groups; equal amounts of capillary connections and sprouts were present. The capillary area was slightly increased in VEGFR-1 TK^−/−^ mice but not significantly suggesting that angiogenic alterations did not induce the differences in LVH development (Figure 5H). Another pathological process during the development of heart failure due to LVH is apoptosis that leads to loss of cardiomyocytes and contractile force. Both groups exhibited similar levels of TUNEL-positive apoptotic cells in the myocardium, suggesting that VEGFR-1 has no role in predisposing cardiomyocytes to apoptosis during LVH (Figure 5I).

### 3.4. VEGFR-1 Levels Are Increased in the Myocardium during LVH 

**Figure 6:**
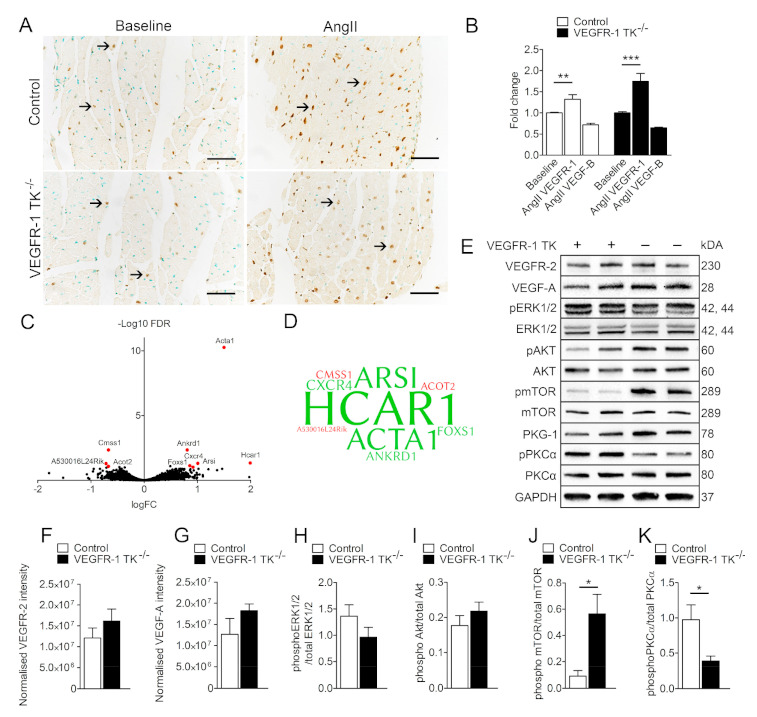
LVH increases VEGFR-1 expression in the myocardium. (**A**) Representative images of VEGFR-1 staining of the myocardium at baseline and after 14-day angII infusion. (**B**) Relative VEGFR-1 and VEGF-B expression measured with RT qPCR (n = 5–7/group). (**C**) RNA sequencing of LVs at day 14 of angII infusion. Volcano plot in which red represents significance in VEGFR-1 TK^−/−^ mice compared to littermate controls with the use of a log fold change (logFC) >0.585 and false discovery rate (FDR) value of <0.10 (n = 4/group). (**D**) Word cloud presentation of differentially regulated genes found in RNA sequencing. Upregulated genes are marked with green and downregulated genes with a red colour. Larger word size correlates with higher fold change (n = 4/group). (**E**) Western blot analysis of LVH signaling cascades at day 14 of angII infusion. Quantification of VEGFR-2 (**F**), VEGF-A (**G**) phosphorylated ERK1/2 (**H**), Akt (**I**), mTOR (**J**) and PKCα (**K**) expression in LVs (n = 4–5/group). Values are mean ± SEM. Statistical analyses were performed using one-way ANOVA and Student’s *t*-test. *** *p* < 0.001; **p < 0.01; * *p* < 0.05.

Next, we stained both intact and LVH myocardium samples for VEGFR-1 to assess protein expression changes during pressure overload. Immunohistological staining revealed that VEGFR-1 levels were increased in hypertrophic hearts compared to baseline. Both VEGFR-1 TK^−/−^ and control mice exhibited increased VEGFR-1 staining and mRNA expression levels in heart during LVH (Figure 6A,B). Furthermore, there was a trend of decreased VEGF-B levels in both VEGFR-1 TK^−/−^ and control mice myocardium after pressure overload.

To investigate the molecular mechanism behind the observed differences in the development of LVH and cardiac dysfunction, we assessed differentially expressed genes in the myocardium of VEGFR-1^−/−^ mice by isolating and deep sequencing cardiac left ventricular RNA after angII infusion. Surprisingly, expression levels were unaltered in isolated cardiomyocytes at the baseline (data not shown) and only 9 genes were significantly regulated in the LVs of angII treated VEGFR-1 TK^−/−^ mice when compared to the controls (Figure 6C,D). Of the differentially regulated genes, 6 were upregulated and 3 downregulated. These data suggest that VEGFR-1 signaling has only minor effects at the transcriptomic level. The most upregulated gene was Hydroxycarboxylic acid receptor 1 (*HCAR1*), which is activated by lactic acid and blockade of HCAR1 may prevent and treat obesity [20]. Novel HCAR1 agonists can also induce hypertension in several species [21]. Another upregulated gene was α-actin 1, encoded by *ACTA1*, which has been shown to be increased in LVH and heart failure both in rodents and in humans [22,23]. In addition, ankyrin repeat domain 1 (*ANKRD1*) expression has been shown to be upregulated in LVs of heart failure patients and it was also upregulated in VEGFR-1 TK^−/−^ LVs [24]. The most interesting upregulated gene however is the chemokine receptor-4 (*CXCR4*), which has been previously shown to be regulated by VEGFR-1 signaling [25,26]. CXCR4 is also upregulated during the development of LVH and AAV9 CXCR4 gene therapy has been shown to prevent adaptive ventricular remodelling after transverse aortic constriction [27].

To further explore the culprits responsible for the observed differences in the development of LVH, we isolated proteins from the LVs after angII infusion and performed Western blot for signaling pathways associated with cardiac hypertrophy and VEGFR-1 signaling. First, we hypothesised that the lack of VEGFR-1 signaling could affect the protein expression levels of VEGFR-2 or VEGF-A in the LV, but we were not able to find any significant changes between the groups for either proteins (Figure 6E–G). Then we looked at the expression levels of proteins important for hypertrophy, such as the protein kinase C (PKC) family of calcium- and/or lipid-activated serine-threonine kinases that have been shown to induce cardiomyocyte hypertrophy and cardiac contractility [28,29]. Our data show that VEGFR-1 TK^−/−^ mice have significantly less phosphorylated PKCα in the myocardium (Figure 6D,H), which is in concordance with reports indicating that disruption of PKCα signaling inhibits pressure overload-induced LVH [30]. Another interesting finding is that the phosphorylation of mammalian target of rapamycin (mTOR) was upregulated more than 5-fold in VEGFR-1 TK^−/−^ mice (Figure 6D,G).

## 4. Discussion

LVH and subsequent heart failure are major health concerns worldwide and especially heart failure has a great impact on both health-care-related costs and patient’s quality of life [2]. Heart failure is a complex syndrome, and its clinical manifestations include breathlessness, fatigue, swelling and cardiac dysfunction. The onset of HFpEF is strongly associated with increasing age, but conditions such as myocardial infarction, hypertension and valvular heart disease are additional risk factors. Unlike HFrEF that has established treatment options to improve the symptoms and the prognosis, HFpEF still remains poorly understood and lacks curative therapies. Although several signaling pathways are known to influence cardiomyocyte hypertrophy and heart failure, the exact mechanisms responsible for pathological cardiac changes in response to pressure overload are yet to be discovered. The revelation of the interaction between cardiac endothelium and myocytes has led to the hypothesis that vascular endothelial growth factors could be important players in the development of LVH and subsequent heart failure [19,31]. VEGF-B is the most abundantly expressed member of the VEGF family in the myocardium and VEGF-B overexpression has been shown to lead to mild hypertrophy and to improve systolic function during LVH after viral transfection [14,32].

In the present study, we show that VEGFR-1 TK signaling is dispensable for normal cardiac function but has an important role in LVH and heart failure. We induced LVH by subcutaneous infusion of angII, which increases the mean arterial pressure within 3 h of implantation of the mini pump [33]. Unexpectedly, VEGFR-1 TK-deficient mice exhibited no LV thickening and unaltered LV volume and EF together with increased mortality at the early time point. Survival curve confirms that the mortality of VEGFR-1 TK^−/−^ mice was the highest on days 3–6 of angII infusion and it coincided with the lack of compensatory LVH and signs of diastolic dysfunction. At later time point, VEGFR-1 TK^−/−^ mice developed a cardiac phenotype characterised by increased ANP and BNP levels and significant cardiomyocyte hypertrophy seen as prolongation of the QRSp duration and increased cardiomyocyte area in histological analysis. Furthermore, the expression of heart failure marker ACTA1 was significantly increased in VEGFR-1 TK^−/−^ mice. All the aforementioned findings suggest that VEGFR-1 signaling is required for the early phase of compensatory LVH, and the ablation of VEGFR-1 TK during pressure overload results in a phenotype similar to human HFpEF.

Reduced angiogenesis and a poor supply of oxygen and nutrients could restrict the development of compensatory LVH and subsequently lead to heart failure. Several studies indicate that VEGFR-1 signaling is important for angiogenesis, but the data lack uniformity since VEGFR-1 TK^−/−^ mice have been shown to express increased CD31-positive vessel area in the myocardium but impaired neovascularisation in the hind limbs [19,25]. To address the role of VEGFR-1 signaling in LVH and heart failure, we stained myocardial capillaries with lectin but were not able to detect any changes in the capillary area or density in VEGFR-1 TK^−/−^ mice when compared to controls during pressure overload. Furthermore, the density of αSMA-positive arteries was similar in both groups. We also performed whole-mount staining for CD31 to visualise the capillary network and CD31-positive vessel area in intact hearts, but again we found no significant differences between the groups. In addition, deep sequencing of cardiac RNA did not reveal expression alterations in genes responsible for angiogenesis or its regulation. Altogether, the data confirm that the delayed development of LVH is not due to alterations in angiogenesis in VEGFR-1 TK^−/−^ mice.

ECG analysis revealed that VEGFR-1 TK^−/−^ mice have significantly increased QRS and QRSp duration at day 14, which is in accordance with the histological finding of enlarged cardiomyocytes. In mice, an increase in QRSp duration might be a more sensitive marker of LV mass increase than QRS complex width [15]. QRS duration is affected both by heart size and impulse conduction velocity, and it has been associated with adverse outcomes in heart failure [15,34]. Also, the amplitude of P wave was found to be significantly increased in VEGFR-1 TK^−/−^ mice, indicating atrial dilatation, a hallmark of heart failure. VEGFR-1 TK^−/−^ mice that did not survive pressure overload had no obvious signs of congestion or heart failure to explain the deaths. The coordination of myocardial excitation-contraction coupling is also disrupted by cardiac fibrosis, characterized by excessive accumulation of extracellular matrix proteins in the myocardium [35]. It is a major hallmark of pathological cardiac remodeling, and it results in the development of scar tissue increasing the stiffness of LV. Interstitial and perivascular fibrosis were evident in both control and VEGFR-1 TK^−/−^ mice at day 14 after angII infusion when assessed from Masson´s trichrome and Picro Sirius Red stainings of the LV sections. However, there were no significant differences between the groups suggesting that VEGFR-1 signaling has no role in the development of fibrosis.

VEGFR-2 and VEGF-A protein expression levels were similar in both control and VEGFR-1 TK^−/−^ myocardium, suggesting that reduced VEGFR-1 signaling is not compensated by other VEGF family members. Also, there were no significant differences in the expression levels of VEGF-B, which has been shown to be downregulated during the progression of LVH [14]. The lack of alterations in VEGF-B expression levels could also account for the absence of metabolic effects in this study. It is interesting that VEGFR-1 mRNA and protein were both upregulated in the LV, emphasising the role of VEGFR-1 signaling in the development of LVH. Several signaling pathways regulate the development of LVH and subsequent transition to a maladaptive phase [36,37]. We found a significant upregulation of cardiac tissue CXCR4 mRNA, which has been shown to prevent adaptive ventricular remodelling after pressure overload [27] and increased ACTA1 levels, that has been shown to be a marker for heart failure [22,23]. We also performed protein analyses which revealed significantly downregulated phosphorylation of PKCα and a 5-fold increase in mTOR phosphorylation in VEGFR-1 TK^−/−^ mice myocardium. We propose that VEGFR-1 downstream signaling regulates several pathways associated with cardiac hypertrophy and the balance of phosphorylation and activity of PKCα and mTOR could inhibit the initial development of compensatory LVH but eventually result in cardiomyocyte hypertrophy at a later time point. Altogether, the data suggest that the lack of VEGFR-1 signaling does not alter the transcriptome extensively but affects the activity of important signaling pathways during pressure overload.

Limitations of our study include the use of a rapidly inducible animal model to mimic human pressure overload in contrast to the typically slow disease progression in humans. Also, the lack of mitral valve doppler echocardiography and measurements of E/e’ ratio does not allow us to thoroughly assess the LV diastolic function, and hence we cannot fully compare our findings to human clinical condition. Furthermore, we were not able to determine the exact cause of death of VEGFR-1 TK^−/−^ mice, which is a general limitation of mouse studies concerning heart failure.

In conclusion, deletion of VEGFR-1 signaling appears not to have an effect on normal cardiac function but it inhibits the development of early compensatory LVH, which in turn leads to cardiac dysfunction associated with atrial dilatation and increased risk of sudden cardiac death, a phenotype somewhat resembling human HFpEF. Eventually, this results in cardiomyocyte hypertrophy mediated by mTOR signaling and increased heart failure markers. We conclude that VEGFR-1 signaling regulates the early remodelling process during the compensatory phase of pressure overload, which might be used as a target for therapeutic interventions of heart failure in the future.

## Data Availability

The RNA sequencing data generated in this publication have been deposited in GEO under accession GSE162489.
